# Evaluation of Associated Lymphomas and Their Risk Factors in Patients with Lymphomatoid Papulosis: A Retrospective Single- Center Study from Turkey

**DOI:** 10.4274/tjh.galenos.2020.2020.0685

**Published:** 2021-02-25

**Authors:** Can Baykal, Sıla Kılıç Sayar, Kurtuluş Didem Yazganoğlu, Nesimi Büyükbabani

**Affiliations:** 1 İstanbul University, İstanbul Faculty of Medicine, Department of Dermatology and Venereology, İstanbul, Turkey; 2 İstanbul University, İstanbul Faculty of Medicine, Department of Pathology, İstanbul, Turkey

**Keywords:** Lymphomatoid papulosis, Mycosis fungoides, CD30+ T-cell lymphoproliferative disorders, Primary cutaneous anaplastic large cell lymphoma, Systemic anaplastic large cell lymphoma

## Abstract

**Objective:**

Lymphomatoid papulosis (LyP) is an indolent skin disease with variable clinical features classified among the primary cutaneous CD30+ T-cell lymphoproliferative disorders. It may show association with cutaneous and systemic lymphomas. We aimed to identify the frequency and characteristics of associated lymphomas among Turkish patients with LyP and to determine the risk factors for secondary lymphomas.

**Materials and Methods:**

The files of patients diagnosed with LyP between 1998 and 2018 in a tertiary dermatology clinic were retrospectively analyzed. Univariate and multivariate models were used to assess the possible risk factors for secondary lymphomas, such as demographic and clinical characteristics of the patients.

**Results:**

Among 61 patients (47 adults, 14 children) with LyP, a total of 22 secondary lymphomas were observed in 20 patients. Nineteen of them were adults. Mycosis fungoides (MF) was the major associated lymphoma (n=19) followed by systemic anaplastic large cell lymphoma (ALCL) (n=2) and primary cutaneous ALCL (n=1). The most common stage in patients with accompanying MF was stage IB (n=11). While 18 patients showed the classical type of MF, one patient had folliculotropic MF. When the risk factors for association between LyP and other lymphomas were evaluated, only older age was found to be a significant risk factor and existence of ulcerated lesions was found to be a negative indicator.

**Conclusion:**

LyP is not rare in the pediatric population. MF is the most common associated lymphoma in patients with LyP. Adult LyP patients are more commonly associated with secondary lymphomas than pediatric patients. Older age at the time of diagnosis of LyP is a significant risk factor for associated lymphomas.

## Abstract (tr)

**Amaç:**

Lenfomatoid papüloz (LeP), primer kutanöz CD30+ T-hücreli lenfoproliferatif hastalıklar arasında sınıflandırılan, değişken klinik özellikler gösterebilen bir deri hastalığıdır. Kutanöz ve sistemik lenfomalarla birliktelik gösterebilir. Bu çalışmada LeP’li Türk hastalarda ikincil lenfomaların sıklığını, özelliklerini ve gelişimleri açısından risk faktörlerini belirlemeyi hedefledik.

**Gereç ve Yöntemler:**

Üçüncü basamak bir dermatoloji kliniğinde 1998-2018 yılları arasında LeP tanısı alan hastaların dosyaları retrospektif olarak incelendi. Hastaların demografik ve klinik özellikleri gibi ikincil lenfomalar açısından risk oluşturabilecek faktörler tek değişkenli ve çok değişkenli istatistik modeller kullanılarak araştırıldı.

**Bulgular:**

LeP’li 61 hastanın (47 yetişkin, 14 çocuk) 20’sinde toplam 22 ikincil lenfoma görüldü. Bu hastalardan 19’u yetişkindi. Mikozis fungoides (MF) en sık görülen ikincil lenfomaydı (n=19) ve onu sistemik anaplastik büyük hücreli lenfoma (ABHL) (n=2) ile primer kutanöz ABHL (n=1) takip ediyordu. Eşlik eden MF hastalarında evre en sık IB (n=11) olarak bulundu. Onsekiz hastada klasik tip MF varken, bir hastada bu kutanöz lenfomanın folikülotropik tipi gözlendi. LeP ve ikincil lenfomalar arasındaki ilişki değerlendirildiğinde, sadece “ileri yaş” anlamlı bir risk faktörü olarak bulundu. “Ülsere lezyon varlığı” ikincil bir lenfoma bulunması için negatif bir gösterge olarak değerlendirildi.

**Sonuç:**

LeP pediatrik popülasyonda nadir değildir. LeP’ye en sık eşlik eden ikincil lenfoma MF’dir. İkincil lenfomalar erişkin LeP hastalarında çocuk hastalara göre daha sık görülür. LeP tanısı sırasında yaşın büyük olması ikincil bir lenfoma için anlamlı bir risk faktörüdür.

## Introduction

Lymphomatoid papulosis (LyP) is a rare indolent disease of the skin that is classified among primary cutaneous CD30+ T-cell lymphoproliferative disorders, alongside primary cutaneous anaplastic large cell lymphoma (pcALCL) [1,2,3], and represents about 12% of cutaneous lymphomas [4]. Clinically it may present with variable numbers of papules, nodules, and tumors, which may ulcerate. Although these lesions heal spontaneously, scars may occur. LyP has an almost excellent prognosis with a 10-year survival rate of up to 100%, with a variable clinical course ranging from a few to frequent relapses [5]. However, this good prognosis may change in patients with associated cutaneous and systemic lymphomas [6,7,8,9,10,11,12,13,14,15,16,17,18,19]. Possible risk factors for secondary lymphomas and the frequency of this association remain unclear [7,9,10,11,15,16,17,18]. In the current study, we aimed to investigate the frequency and characteristics of the associated lymphomas in our large series of patients with LyP and to determine the potential risk factors for secondary lymphomas among Turkish LyP patients.

## Materials and Methods

### Patients and Setting

This retrospective study was conducted  in the Department of Dermatology and Venereology of the İstanbul Faculty of Medicine, a tertiary referral center with a subspeciality clinic for primary cutaneous lymphomas in Turkey, and it was approved by the institutional ethics committee. We reviewed the data of 61 patients consecutively diagnosed with LyP between 1998 and 2018. Diagnosis was established with clinicopathological correlation. Patients’ files were evaluated regarding demographic features (i.e. sex, age), clinical presentation of LyP (i.e. localized/generalized, existence of ulcerated lesions), histological subtypes of LyP, and accompanying cutaneous lymphomas (i.e. type, subtype, and stage) or systemic lymphomas (i.e. type and chronology). The diagnosis of accompanying mycosis fungoides (MF) was based on clinical features and confirmed by histopathologic evaluation in all cases. Patients younger than 18 years were accepted as pediatric cases.

In addition to the physical examination including checking for peripheral lymph nodes, patients underwent routine blood testing and basic full-body imaging studies at the time of diagnosis. Routine blood tests were repeated at least once per year for patients during the follow-up period. The diagnosis of accompanying systemic lymphomas was based on hematologic examination.

### Statistical Analysis

Statistical analysis was performed using SPSS 22 (IBM Corp., Armonk, NY, USA). Student’s t-test was used to compare  continuous variables. The chi-square test and Fisher’s exact test were used to compare categorical variables between the two groups of patients with LyP (with and without secondary lymphomas). The variables with a p-value of less than 0.10 in the univariate analysis were incorporated into the multivariate regression model. A p-value of less than 0.05 was considered statistically significant for all tests.

## Results

### Demographic and Clinical Characteristics of the Patients and Histological Subtypes of LyP

**Figure 1:**
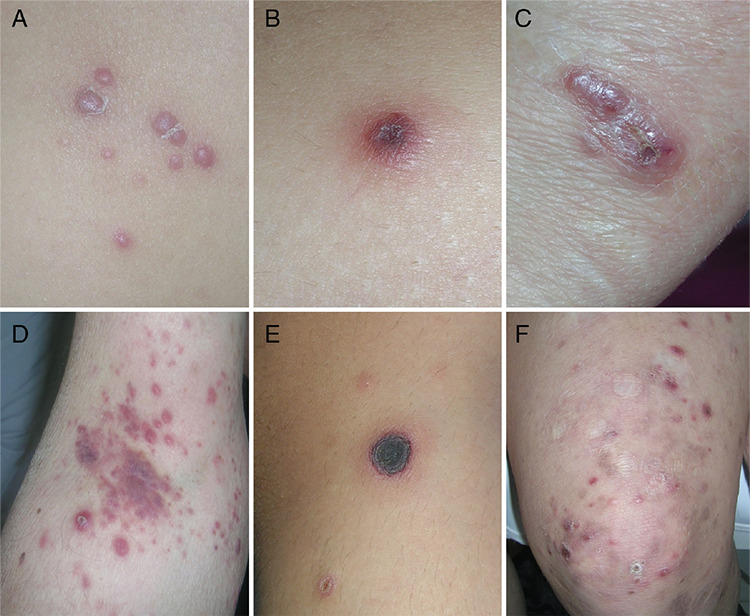
Lesions of lymphomatoid papulosis: **A)** Multiple papules. **B)** Isolated hemorrhagic papule. **C)** Nodule. **D)** Agminated papules and nodules. **E)** Ulcerated nodule. **F)** Papules and nodules associated with atrophic scars of former lesions.

**Figure 2:**
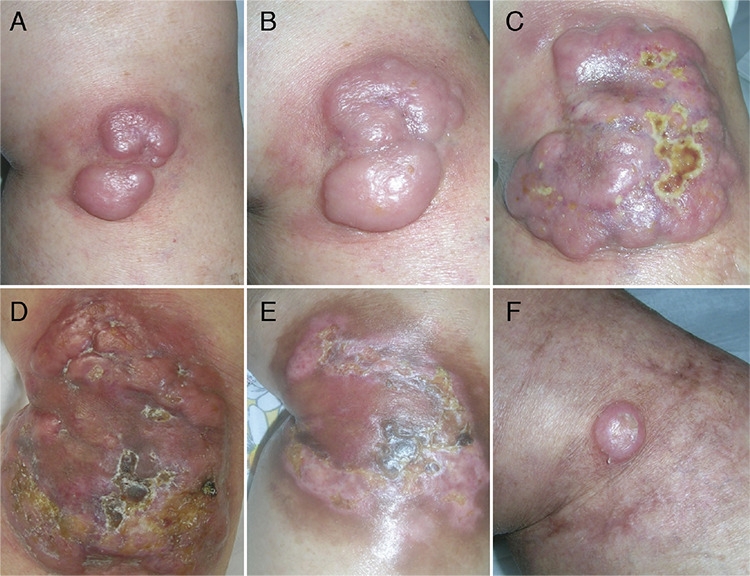
Evolution of a tumor of lymphomatoid papulosis (Patient 14 in Table 2). **A-C)** Enlargement and confluence of two nodules forming one large tumor in two weeks. **D, E)** Flattening of the tumor followed by spontaneous healing in three weeks. **F)** Relapse of a nodule at the same site one year later.

Sixty-one patients consisting of 31 females (50.8%) and 30 males (49.2%), who were diagnosed with LyP over a 21-year period, were included in the study. The mean ages at the time of onset and at the time of diagnosis were determined as 33.5±17.2 years (median: 32; range: 2-76 years) and 36.4±18.1 years (median: 36; range: 5-76 years), respectively. The mean age of the pediatric patients (n=14, 23%) at the time of disease onset was 9.7±4.2 years (range: 2-17 years). The mean time lapse of symptoms prior to diagnosis in all patients was calculated as 32.9±58.7 months (median: 12; range: 1-288 months). The mean follow-up period from admission to the last visit was 5.6±6.7 years (median: 3; range: 1-21 years). Table 1 summarizes the demographic and clinical characteristics of our study group. While all patients had cutaneous involvement such as papules, nodules (Figures 1a-1f), or tumors (Figure 2) mostly associated with scars (Figure 1f), an adult patient had accompanying mucosal lesions, as well. Lesions were disseminated in 51 patients, whereas 10 adults had localized lesions (Figure 1d). One or more ulcerated lesions (Figure 1e) were seen in 14 patients.

The histological subtypes according to the WHO-EORTC classification could be determined in 26 of the patients (Table 1);  type A (n=9) was the most common, followed by type C (n=7), type B (n=5), type D (n=2), and type E (n=1). The remaining two patients had unusual combined histologic features of types A and C. In the other 35 patients a specific type could not be assigned based solely on histopathologic findings. Differentiation of LyP from pcALCL was based on the clinicopathological features and course of the disease.

### Associated Lymphomas in Patients with LyP

A total of 22 associated lymphomas were detected in 20 LyP patients (32.8%) consisting of 8 males and 12 females. Accompanying lymphomas were MF in 17 patients, MF and systemic anaplastic large cell lymphoma (sALCL) in two patients, and pcALCL in one patient (Table 2). MF was the most common accompanying lymphoma (n=19; 31.1%) in all LyP patients, and it constituted 86.4% of all associated lymphomas (19 out of 22) in our series.

The diagnosis of LyP was made concurrently with the diagnosis of MF in 1 patient, following the diagnosis of MF in 14 patients, and prior to the diagnosis of MF in 4 patients (Table 2). Both sALCL and pcALCL occurred after the diagnosis of LyP in 3 patients.

Among the 14 patients who had MF previously, the mean time between the diagnosis of MF and the diagnosis of LyP was 5.9 years (range: 0.5-20 years). On the other hand, MF developed in a mean duration of 4.3 years (range: 1-10 years) after the diagnosis of LyP in 4 patients.

While 18 patients showed the classical type of MF, the remaining patient had the folliculotropic type. The most common stage in accompanying MF patients was stage IB (n=11), followed by stages IA (n=5) and IIA (n=2). One patient had stage IVA MF (neoplastic lymph node infiltration).

Only one pediatric LyP patient had a secondary lymphoma (stage IA MF), while the rest (n=13) had no sign of secondary lymphoma in a mean follow-up duration of 4.8±4 years (median: 3 years).

### Assessment of Risk Factors for Associated Lymphomas

While older age at the time of diagnosis was found to be a significant risk factor for associated lymphomas among LyP patients in univariate analysis (p=0.03), the existence of ulcerated lesions was found to be a negative indicator of a secondary lymphoma (p=0.02) (Table 3). Sex of the patients and distribution of the lesions (localized or generalized eruption) did not have a statistically significant association with secondary lymphomas (Table 3). On the other hand, the association between older age and secondary lymphomas was significant in multivariate analysis (OR: 1.05; 95% CI: 1.01-1.08; p=0.03) (Table 3). While existence of ulcerated lesions was found to be a significant negative indicator of secondary lymphomas (OR: 0.07; 95% CI: 0.01-0.99; p=0.03), the rest of the clinical variables (distributions of lesions) were not associated with secondary lymphomas in the multivariate model (Table 3).

Among histopathologic subtypes of LyP, type C showed the highest association with secondary lymphomas (5 out of 7 cases; 71.4%), followed by type B (2 out of 5 cases; 40%) and type A (2 out of 9 cases; 22.2%) (Table 2). Further statistical analysis to determine which histological subtype could contribute as a risk factor for associated lymphomas could not be done due to the missing data of histological subtypes in many cases.

### Survival and Prognosis

Three (4.9%) patients in our series have died to date, due to the complications of systemic ALCL in two cases and a cause unrelated to LyP in the remaining patient without a lymphoma association. No progression in the stage was observed among MF patients (n=14) in whom LyP occurred afterwards in a relatively long follow-up period (mean: 5.9 years).

## Discussion

Being the largest study conducted in Turkey on LyP patients, this study highlights some similarities as well as some differences from previous reports [6,7,8,9,10,11,12,13,14,15,16,17,18]. While many studies on the demographics of LyP, including the largest series reported from the Netherlands [6], determined a male predominance [6,7,8,9,10,12,13,14,15], the present study shows near equality between the sexes with a slight female predominance. The mean age at diagnosis of these 61 LyP patients was 36.4 years, which is a little younger than that reported in previous studies that included adult and pediatric patients [6,8,12,13,15,16,17,18]. This situation may be caused by the fact that Turkey has a relatively young population. Moreover, more than one-fifth of the patients (23%) in our study group were younger than 18 years. LyP is generally known to be less frequent among pediatric patients compared to adults [20,21]. Although some reports from the Netherlands and United States showed considerable rates of pediatric patients (<20 years old) of up to 10% in their series of LyP cases [6,15,17], our results exhibited a much higher percentage of pediatric patients (<18 years old). The diagnosis of LyP is probably overlooked or misdiagnosed among the pediatric population due to the limited number of lesions in some patients, reluctance of the parents to permit biopsies, and the benign course of the disease with spontaneous resolution [22].

While histological subtypes could be exactly determined in less than half of our group, the frequencies were similar to those of other reports, with type A being the most common, followed by types B and C [10,11,15,17]. Type A has usually been found to be the most common histological LyP subtype in the literature, and it is less frequently associated with secondary lymphomas compared to the other types [7,10,11,17]. In the present study, type C showed the highest association with secondary lymphomas (71.4%), followed by type B (40%) and type A (22.2%) (Table 2); however, we could not further analyze which histological subtype could contribute as a risk factor for associated lymphomas due to the missing data of histological subtypes in many cases. On the other hand, some previously reported retrospective studies assessing a certain histological subtype being a risk factor for secondary lymphomas may not always reflect a true association due to the recent description of some other histologic subtypes (i.e. D, E, and F) that were not known at the time of these studies because of their retrospective design [9,10].

**Figure 3:**
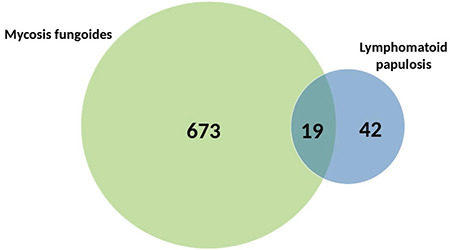
The distribution of MF and LyP cases diagnosed between 1998 and 2018 in our clinic.

Almost one-third of the patients (n=20, 32.8%) in the current study had secondary lymphomas including MF (n=19), sALCL (n=2), and pcALCL (n=1) (2 patients had both MF and sALCL). This rate was within the range of the rates previously reported, varying from 6% to 64% [6,7,8,9,10,11,12,13,14,15,16,17,18]. However, the frequency of MF in LyP patients (31.1%) was strikingly high in our series with a percentage of 86.4% among all secondary lymphomas, exhibiting one of the highest associations of MF with LyP when compared with formerly reported studies stating rates of 5% to 39% for the patients having this cutaneous T-cell lymphoma in their series [6,7,8,9,10,11,12,15,16,17,18]. This may be explained by a referral bias due to our subspecialty clinic being dedicated to primary cutaneous lymphomas and especially focused on MF. A total of 692 patients were diagnosed with MF during the same 21 years in our clinic (unpublished data), and the ratio of association of LyP in this MF group was 2.7% (n=19) (Figure 3). In a recently reported large series of MF patients (n=580), the rate for association of LyP was found to be 1.9% [19]. Interestingly, in the same study, a significant association of LyP among Caucasian MF patients (3%) was reported when compared to African American MF patients (0%) [19].

The relationship of LyP with the subtypes of MF has not been well documented in the literature. In the present study, most LyP-MF associated cases had the classical presentation of MF except one patient with the folliculotropic subtype. It has been proposed that MF cases associated with a primary cutaneous CD30+ T-cell lymphoproliferative disorder seem to have an indolent clinical evolution and a favorable prognosis [23]. Likewise, most of the MF patients in this study were in early stages (patches and/or plaques) of the disease. Neoplastic lymph node infiltration was only seen in one case. Moreover, progression of the MF stage was not observed among the 14 patients in whom LyP occurred afterwards. Three patients (4.9%) in our series died during the 21 years of the study period, two of them due to complications of sALCL and the other, without secondary lymphoma, from a cause unrelated to LyP.

Systemic lymphomas (mostly sALCL and Hodgkin’s lymphoma and rarely others such as chronic lymphocytic leukemia, non-Hodgkin lymphoma, and myelodysplastic syndrome) in association with LyP were reported at variable rates from 1.1% to 13.2% [6,7,9,10,11,12,15,16,17,18]. We have observed only two cases of sALCL (3.3%). On the other hand, one case of pcALCL (1.6%) was observed in our series, in contrast to some previous studies stating remarkable amounts of LyP cases associated with pcALCL at up to 20% (range: 1.5%-20%) [6,7,9,10,11,12,15,16,17,18]. Since the histological appearances of LyP and pcALCL have many features in common, the differential diagnosis between these two diseases usually depends on the clinical decision, particularly based on the course of the lesions, and it is usually a matter of debate. Indeed, in some of our cases we observed large nodules or tumors resembling pcALCL, but spontaneous resolution of these lesions in a short time supported the diagnosis of LyP (Figure 2). Therefore, the high rates of this association in some studies may be due to misinterpretation.

A systematic review including 251 children (aged 18 or younger at the time of diagnosis) with LyP reported a rate of 5.6% (n=14) for secondary lymphomas including mostly pcALCL (n=10). MF (n=1), sALCL (n=2), and an atypical cerebellar lesion that had the same T-cell clonality as LyP (n=1) represented other associations [20]. In our study, only one child had a secondary cutaneous lymphoma (MF), and the remaining 13 pediatric patients did not develop any secondary lymphomas in the short follow-up period (mean: 4.8±4 years; median: 3 years).

A variety of risk factors for associated lymphomas among LyP patients have been reported so far, including male sex [7,10,16], B and C histological subtypes of the disorder [10,11], presence of a T-cell clone [7,9,15], frequent recurrences [11], younger age at onset [11] and older age at diagnosis [7,9]. In addition, rarely investigated variables such as exposure to Epstein-Barr virus [16] and existence of head lesions [11] were found to be significant risk factors. In our study, only older age at diagnosis was found to be a significant risk factor for associated lymphomas, similar to some of the previous studies [7,9]. On the other hand, the existence of any ulcerated lesion was highlighted here as a possible negative indicator of secondary lymphomas, which was not reported formerly. Ulceration does not have a known prognostic significance in LyP and this result needs further confirmation in other series.

### Study Limitations

The main limitation of this study was its retrospective design.

## Conclusion

More than one-fifth of the LyP patients in our series were pediatric patients (23%), which underlines the importance of considering LyP in the differential diagnosis of such cutaneous lesions among the pediatric population. Almost one-third of the LyP patients had accompanying MF (31.1%), which points out a high association of LyP with MF. On the contrary, pcALCL was not common in our series, causing us to question the importance of this association. Adult LyP patients are more commonly associated with secondary lymphomas than pediatric patients. The only risk factor for associated lymphomas in LyP patients was found to be older age in our study. Therefore, it is essential to closely follow adult patients with LyP.

## Figures and Tables

**Table 1:**
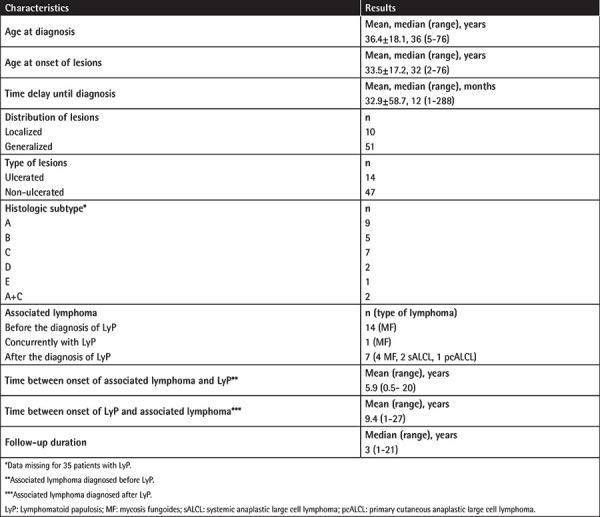
Demographic, clinical, and histological characteristics of 61 patients with LyP.

**Table 2:**
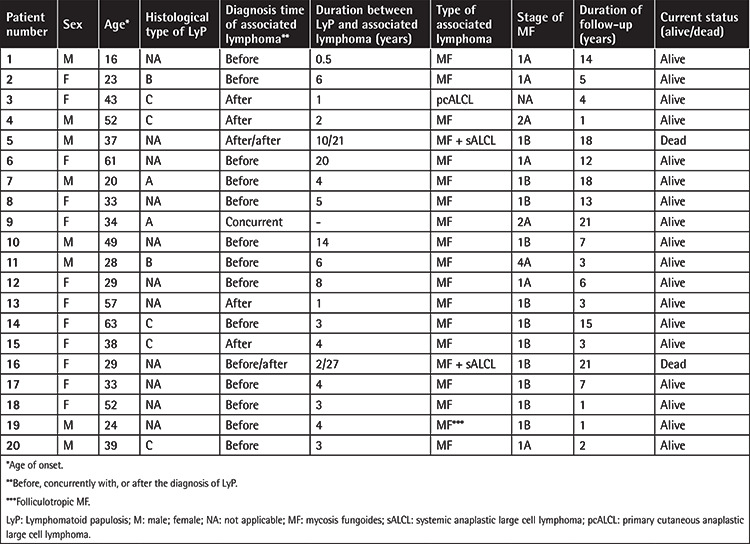
General characteristics of 20 patients with LyP-associated lymphomas.

**Table 3:**
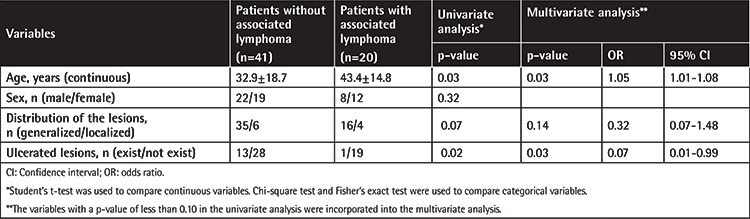
Univariate and multivariate analysis of variables for association of secondary lymphomas in patients with lymphomatoid papulosis.
